# Development of High-Efficiency Perovskite Solar Cells and Their Integration with Machine Learning

**DOI:** 10.3390/nano15211608

**Published:** 2025-10-22

**Authors:** Shihao Gao, Ruowen Peng, Kuankuan Ren, Lina Yu, Qi Jiang, Zhanwei Shen, Shizhong Yue, Zhijie Wang

**Affiliations:** 1 Laboratory of Solid-State Optoelectronics Information Technology, Institute of Semiconductors, Chinese Academy of Sciences, Beijing 100083, China; gaoshihao25@mails.ucas.ac.cn (S.G.); pengruowen25@mails.ucas.ac.cn (R.P.); yulina@semi.ac.cn (L.Y.); qjiang@semi.ac.cn (Q.J.); zwshen@semi.ac.cn (Z.S.); wangzj@semi.ac.cn (Z.W.); 2 Zhejiang Engineering Research Center of MEMS, School of Mathematical Information, Shaoxing University, Shaoxing 312000, China; 3 AnnLab, Institute of Semiconductors, Chinese Academy of Sciences, Beijing 100083, China; 4 State Key Laboratory of Semiconductor Physics and Chip Technologies, Institute of Semiconductors, Chinese Academy of Sciences, Beijing 100083, China

**Keywords:** perovskite solar cells, power conversion efficiency, stability, lead-free, machine learning

## Abstract

Perovskite solar cells, as a rising star in third-generation photovoltaic technologies, have attracted extensive attention due to their high light absorption, tunable bandgap, and high power conversion efficiency, indicating substantial potential for future applications. Starting from the development history of perovskite solar cells, this review systematically comprehends the technological breakthroughs in the continuous improvement of power conversion efficiency since their invention, outlining the research status and technical bottlenecks. A detailed analysis is provided on the material characteristics and limitations of the lead-based perovskite systems. Critical obstacles towards commercialization are also identified, such as operational instability and the challenges associated with large-scale manufacturing. Finally, the potential role of machine learning in the discovery and design of new perovskite materials is highlighted, and future development directions have been outlined. Special focus is placed on the innovative applications of machine learning in material composition screening, material properties prediction, and process parameter optimization, with the aim of constructing a closed-loop research framework. The review aims to offer valuable insights and references for advancing the performance and industrial applications of perovskite solar cells.

## 1. Introduction

Against the backdrop of the global energy structure transitioning to low carbonization, innovation in solar energy utilization technologies is crucial. Since their advent in 2009, perovskite solar cells (PSCs) have demonstrated extraordinary development potential with an ultra-high power conversion efficiency (PCE) improvement rate. Generally, PSCs consist of a perovskite light-absorbing layer, electron transport layers (ETL), and hole transport layers (HTL). Perovskite materials possess a direct bandgap with adjustable width. This characteristic enables the light-absorbing layer to absorb light across a broader spectral range, thereby endowing perovskite solar cells (PSCs) with excellent photovoltaic conversion efficiency [1]. Meanwhile, their strong weak-light absorption capability also opens up opportunities for their application in indoor light environments [2]. Additionally, PSCs have become a research hotspot in recent years due to their unique advantages, including simple fabrication processes and low costs.

The main challenge currently facing PSCs towards commercialization is their stability, and the practical contradiction lies in the gap between laboratory efficiency breakthroughs and industrialization. This is reflected in the PCE degradation of large-area PSCs and insufficient long-term stability. Studies have shown that when the area of PSCs is scaled up to practical sizes, their PCE decreases significantly [3]. Moreover, perovskite films are susceptible to thermal stress-induced irreversible phenomena such as decomposition and film peeling, severely restricting device performance. Although the stability of small-area PSCs has been significantly improved through methods such as grain boundary modification, interface engineering, and encapsulation technologies, the stability issue of large-area perovskite modules remains unsolved [4,5,6].

Considering the wide variety of PSCs, selecting appropriate types and device structures can provide directions for the development of PSCs. The cross-integration of machine learning (ML) and perovskite research is offering a new path to address these challenges—through building a “data-model-optimization” closed loop, ML has achieved a 100-fold improvement in screening efficiency for lead-free perovskite materials and a 50% expansion in the process window for thin-film fabrication, demonstrating enormous potential for interdisciplinary innovation [7]. Current research on PSCs focuses on the development of new materials, large-area fabrication of devices, flexible perovskite devices, and their tandem integration with other devices.

This review systematically outlines key research progress in PSCs, providing a comprehensive analysis and synthesis of various research approaches related to the structural evolution of perovskite layer, device design of both single-junction and tandem/multijunction devices, and methods for improving device stability. It also discusses the industrialization progress of PSCs and their development trends in performance improvement and large-area fabrication. Subsequently, with the goal of integrating ML into PSCs research, this study comprehensively evaluates methods and strategies for performance improvement from various aspects, including high-throughput screening of material compositions, optimization of fabrication techniques, refinement of processing methods, and predicting product performance, to obtain PSCs with higher efficiency, simplified production, and better stability. Furthermore, some studies focus on elucidating the relationship between process parameters and materials properties, shortening optimization time, and providing innovative pathways for the application of ML in perovskite material research and development. Thus, this review also offers an in-depth analysis of the mechanisms underlying power conversion efficiency and stability degradation, alongside a discussion of potential countermeasures. Finally, it prospects future research directions of PSCs, aiming to provide theoretical insights and technical guidance to advance the performance improvement and industrialization of PSCs.

## 2. Material and Device Structure

### 2.1. Material Structure

**Figure 1:**
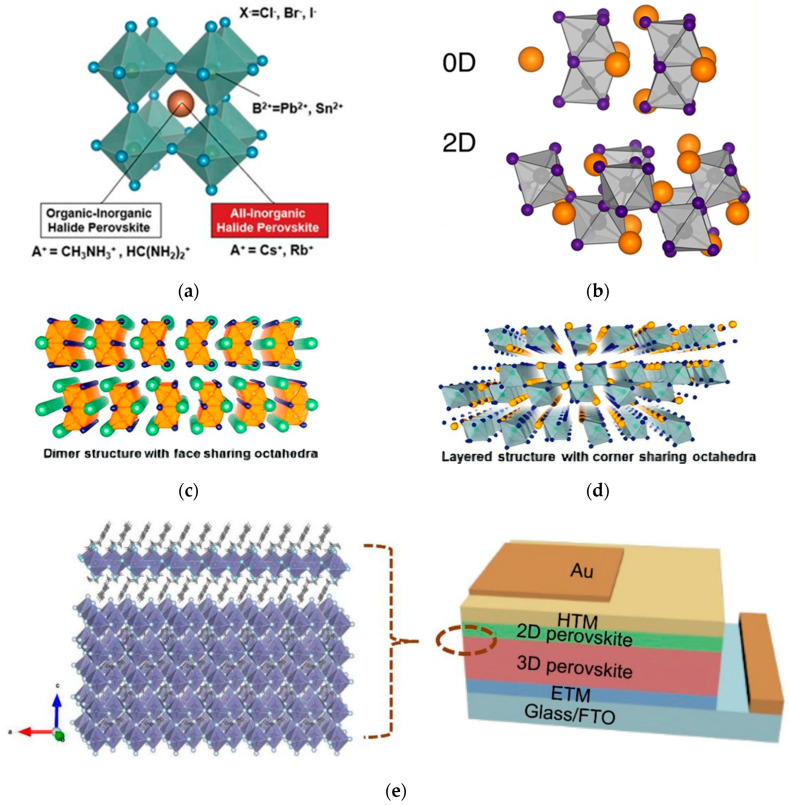
Structural evolution of PSCs. (**a**) Schematic diagram of 3D perovskite structure [9] Copyright 2017, Advanced Science. (**b**) Schematic diagram of 0D/2D perovskite structure [10] Copyright 2015, American Chemical Society. (**c**) Schematic diagram of dimer structure with face-sharing octahedra. (**d**) Schematic diagram of 0D perovskite structure [11] Copyright 2016, American Chemical Society. (**e**) Schematic representation of 2D/3D perovskite device [12] Copyright 2018, Advanced Functional Materials.

Perovskite was originally the name of the mineral CaTiO_3_, named after the Russian geologist Perovski [8]. In a narrow sense, perovskite refers to the mineral CaTiO_3_ itself, while in a broad sense, it refers to ABX_3_-type compounds with an octahedral structure, where A is a monovalent organic or inorganic cation (usually methylammonium cation (MA^+^), formamidinium (FA^+^), cesium ion (Cs^+^), etc.), B is a divalent metal cation (usually Pb^2+^, Sn^2+^, Bi^2+^, etc.), and X is a monovalent halogen anion (usually F^−^, Br^−^, Cl^−^, I^−^, etc.) [8]. Its structures generally include simple perovskite structure, double perovskite structure, and layered perovskite structure, similar to anorthite; from the perspective of structural dimensionality, it can also be divided into three-dimensional (3D), two-dimensional (2D), and zero-dimensional (0D) perovskite materials [9] (structures shown in Figure 1a–d). Compared with 3D perovskites, low-dimensional perovskites (2D, 0D) exhibit unique properties, such as diverse crystal structures, low ion mobility, and good stability, whose properties can be adjusted through spacer organic cations [10,11]. The composite structure formed by the combination of 2D perovskites and 3D perovskites enables the complementation and synergistic optimization of the properties of the two-dimensional perovskite materials [11]; the device structures are shown in Figure 1e.

**Figure 2:**
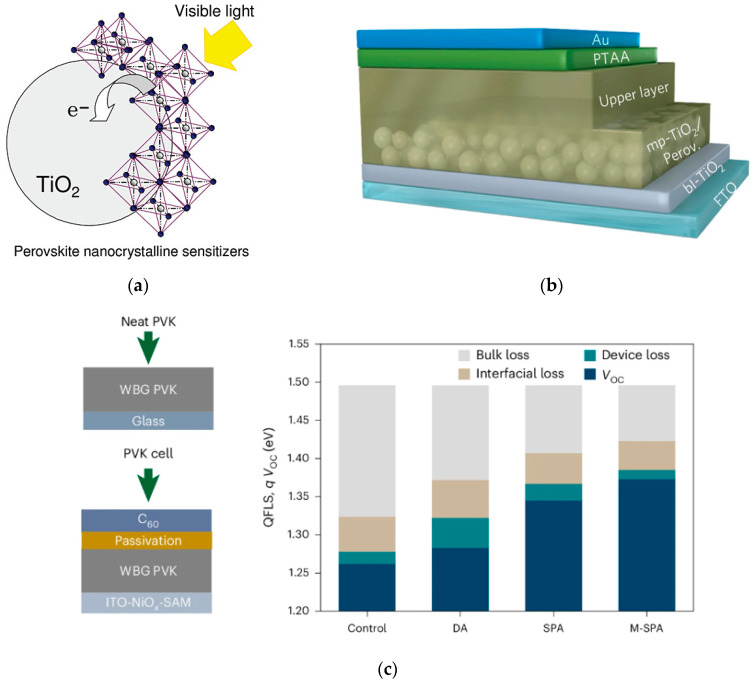
Typical perovskite structure. (**a**) First application of perovskite cells [12] Copyright 2009, Journal of the American Chemical Society. (**b**) The first mesoporous perovskite solar cell structure [13] Copyright 2014, Nature Mater. (**c**) Schematic diagram of perovskite films and device structures for measurement, as well as the obtained device performance diagram [14] Copyright 2025, Nature Mater.

As a rising star in third-generation solar cells, PSCs show broad application prospects due to their high light absorption, tunable bandgap, and high PCE. In 2009, Miyasaka et al. applied methylammonium lead iodide (CH_3_NH_3_PbI_3_) to quantum dot-sensitized solar cells (as shown in Figure 2) [13], fabricating the first PSC with an efficiency of 3.8%. Despite still facing issues such as chemical and environmental instability, this marked the beginning of the research journey for perovskite solar cells. In 2011, Park designed the first mesoporous PSC [14], increasing the efficiency to 6.5%. Since then, PSC research has been divided into two main structures: mesoporous and planar. Currently, advanced perovskite solar cells include a 1.05 cm^2^ all-perovskite tandem solar cell fabricated by the team of Tan with a steady-state PCE of 28.2% [15], demonstrating the great potential of tandem devices. Opto-Electronics Technology developed an all-perovskite tandem solar cell with a PCE of 31.27%, setting a new world record [16]. A team of researchers from the Helmholtz Center and Humboldt University in Germany has developed a new type of stacked solar cell by combining a copper indium gallium selenide (CIGS) cell for the bottom with a top cell based on perovskite. The highest certified efficiency of single-junction PSCs listed in the NREL efficiency chart is 26.95% [17], maintained by the team of Peng and Zhang from Soochow University. The highest record for all-perovskite tandem cells is 30.1%, developed by the team of Tan from Nanjing University in collaboration with Renshaw Solar Energy. Obviously, within less than two decades, the PCE of PSCs has been significantly improved, making them considered as the most promising next-generation solar cells.

The general formula of ideal perovskite is ABX_3_, with a cubic crystal structure. The ABX_3_ structure can be approximately regarded as a result of close packing, with the packing layers perpendicular to the body diagonal (111) of the cube. Here, A and X are indistinguishable and arranged in a cubic close-packed manner, while the smaller cation B fills the octahedral gaps.

**Figure 3:**
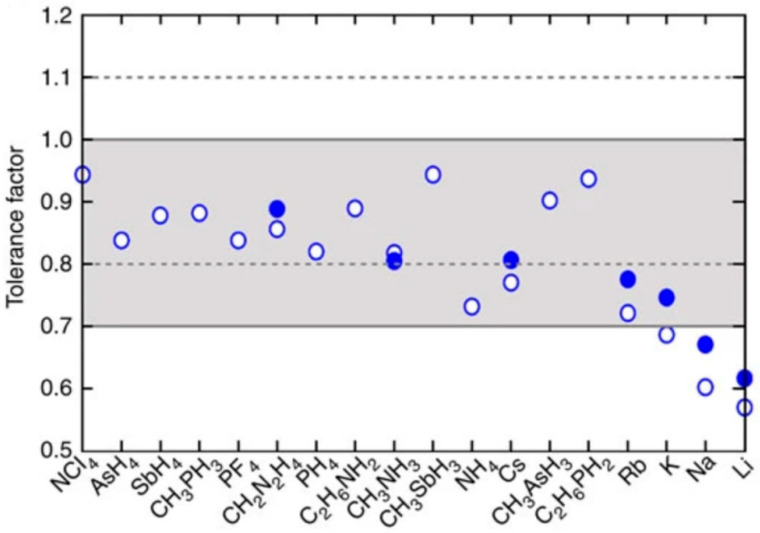
Tolerance factors of various cations in perovskite structures [18] Copyright 2014, Nature Communications.

From the perspective of coordination polyhedra, the crystal structure of the ideal perovskite ABX_3_ can be regarded as a structure formed by multiple [BX_6_] octahedra connected through sharing vertices in three-dimensional space. This ideal structure is called the prototype of perovskite. Under the action of external forces, a series of distortions will occur in the ideal structure to form low-symmetry structures, such as tetragonal, orthorhombic crystal systems, etc. Such a structure is then called the heterotypic structure of perovskite. In order to better describe the stability of the perovskite structure, the “tolerance factor” t is introduced to evaluate this relationship quantitatively [18], that is,

(1) $$t=(R_{A}+R_{X})/\sqrt{2}(R_{B}+R_{X})$$

where R_A_, R_B_, and R_X_ represent the radii of A-site cations, B-site cations, and X-site anions, respectively. When multiple ions are present at the A or B site, their average radius is used. Generally, when t is close to 1.0, the perovskite crystal has high symmetry, usually belonging to the cubic system; when t deviates significantly from 1.0, other low-symmetry structures are usually formed [7]. The tolerance factors of various cations in perovskite structures are illustrated in Figure 3.

### 2.2. Device Structure of PSCs

**Figure 4:**
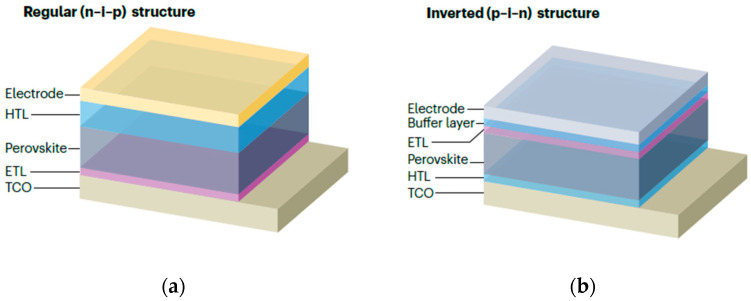
Typical perovskite solar cells structures. (**a**) Regular (n-i-p) structure; (**b**) inverted (p-i-n) structure [20] Copyright 2024, Springer Nature.

The basic structure of PSCs is sandwich-like, consisting of a substrate, electrodes, charge transport layers, and a perovskite light-absorbing layer. Different types of perovskite devices vary in the position and material of the ETL [19]. The main types of structures are shown in Figure 4. Planar heterojunction devices can be divided into normal (nip) and inverted (pin) structures according to the position of the charge transport layer [20].

In the normal structure, the electron transport layer is adjacent to the metal electrode, and the HTL is adjacent to the transparent electrode. Common ETL materials include TiO_2_, SnO_2_, etc., and HTL materials such as Spiro-OMeTAD. The advantage of the normal structure is that the electron transport layer can be fabricated at low temperatures, which not only significantly saves energy and also reduces costs, but it has a high hysteresis effect. The inverted structure is one in which the positions of the ETL and HTL are swapped. This structure has high stability, simple fabrication methods, and an almost negligible hysteresis effect. Commonly used HTL materials include PEDOT:PSS, PTAA, and ETL materials include PCB, C_60_, etc. According to the presence or absence of a mesoscopic structure, they can be divided into mesoporous PSCs and planar heterojunction PSCs. The main function of the mesoporous layer is to provide a supporting framework to limit the growth of perovskite crystals. The Grätzel team developed a mesoporous structure in the early stages, increasing the efficiency of PSCs from 3.8% to over 15% [21].

At present, the commonly used ETL materials are mainly categorized into two major types: oxide semiconductors and organic polymers. Among them, perovskite oxides have exhibited broad application prospects in PSCs due to their excellent electrical properties, thermal stability, and environmental friendliness. An ideal ETL material should possess a conduction band position that matches the perovskite light-absorbing layer, high electron mobility, low trap state density, and good thermal stability. Currently, the most extensively studied perovskite oxide ETL materials include TiO_2_, SnO_2_, and ZnO. Specifically, TiO_2_ was the earliest to be applied but has moderate electron transport performance; SnO_2_ demonstrates excellent electron transport properties; ZnO offers advantages such as stable characteristics and simple preparation, yet it has poor chemical compatibility with perovskite materials. A variety of targeted modification strategies have also been developed. For instance, Xian et al. incorporate metal Ga into the TiO_2_ precursor solution, successfully fabricating a TiO2-Ga layer. The TiO_2_ electron transport layer doped with Ga can effectively improve the PCE of PSCs [22]; Fang et al. prepared effective lignin carbon dot-based dopants and passivators, which mitigate the defects of SnO_2_ and improve the performance of PSCs [23].

The construction of bilayer or multilayer ETL structures, such as TiO_2_/SnO_2_ and TiO_2_/ZnO, has also been proven to be an effective approach for improving electron transport efficiency and suppressing interfacial recombination. In addition, there are structural types lacking an electron transport layer or a HTL. The ETL-free structure can utilize the intrinsic bipolar semiconductor properties of perovskite itself, reducing issues caused by the electron transport layer, but it faces challenges such as low open-circuit voltage. In addition, Sajid et al. [24] utilized diethanolamine (DEA) to create a favorable dipole layer at the interface between the perovskite layer and indium tin oxide (ITO) substrate through molecular adsorption. This dipole layer effectively suppressed interfacial charge recombination. PSCs fabricated on DEA-treated ITO substrates achieved a maximum PCE of 20.77%, which ranks among the highest efficiencies reported for ESL-free PSCs.

Perovskite oxides, serving as HTLs, play a crucial role in PSCs with a p-i-n structure. An ideal HTL material should possess a valence band position that matches the perovskite light-absorbing layer, high hole mobility, and excellent thermal stability. Currently, the widely investigated perovskite oxide HTL materials include NiO_x_, copper oxides (CuO/Cu_2_O), and copper-based oxides (e.g., CuGaO_2_, CuAlO_2_).

NiO_x_ is one of the most extensively studied inorganic hole transport materials, featuring a large bandgap (approximately 3.6 eV) and a deep valence band maximum (approximately 5.4 eV), which enables excellent energy level matching with perovskite materials. Han et al. [25] developed p-type heavily doped Ni_0.8_Li_0.05_Mg_0.15_O (NiLiMgO), which significantly enhances the electrical conductivity and carrier concentration of NiO_x_.

### 2.3. Comparative Analysis of Different Thin-Film Preparation Technologies

Spin-coating is one of the most commonly used methods for preparing perovskite thin films. Its basic principle involves dropping a solution onto a rotating substrate, where centrifugal force drives the uniform spreading of the solution to form a thin film. Spin-coating offers advantages such as simple operation, low equipment cost, high-quality film formation, and good reproducibility, making it the most widely employed preparation method in laboratory research.

Thermal evaporation is a physical vapor deposition (PVD) technique. It involves heating the source material in a vacuum environment to induce evaporation, and the vaporized material is then deposited onto the substrate to form a thin film. In PSCs, thermal evaporation is mainly used for fabricating metal electrodes and some functional layers. For instance, when preparing the SnO_2_ ETL, SnO_2_ thin films can be deposited on the substrate via thermal evaporation.

Sputtering is another commonly used PVD technique. In a vacuum environment, high-energy ions are utilized to bombard the target material, causing atoms or molecules of the target to be ejected and deposited onto the substrate to form a thin film. In addition, technologies such as sputtering, atomic layer deposition (ALD), and solution processing each have their own advantages and disadvantages: Spin-coating features simple operation and high-quality film formation, making it suitable for laboratory research and small-scale production; thermal evaporation and sputtering can produce high-quality thin films, but they suffer from high equipment costs, which render them unsuitable for large-scale production; ALD enables atomic-level thickness control and excellent uniformity, yet it is limited by high equipment costs and low production efficiency; solution processing technologies, particularly blade coating and inkjet printing, possess high production efficiency and low cost, thus being suitable for large-scale production.

### 2.4. All-Perovskite Tandem Solar Cells

All-perovskite tandem solar cells consist of a narrow-bandgap bottom cell and a wide-bandgap top cell, with the advantage of significantly reducing optical losses and efficiently capturing photons in the solar spectrum. Their working principle is based on the absorption and conversion of light of different wavelengths in the solar spectrum by perovskite materials with different bandgaps, enabling better matching between photon energy and material bandgap, and reducing thermal losses caused by photon energy higher than the material bandgap. The characteristic of all-perovskite tandem cells is that the bandgaps of the two sub-cells can be flexibly adjusted, maximizing the efficient utilization of the solar spectrum, reducing optical losses, and increasing the open-circuit voltage (V_oc_) of the device, thus greatly improving the PCE, which is expected to break through the Shockley–Queisser efficiency theoretical limit of single-junction solar cells.

**Figure 5:**
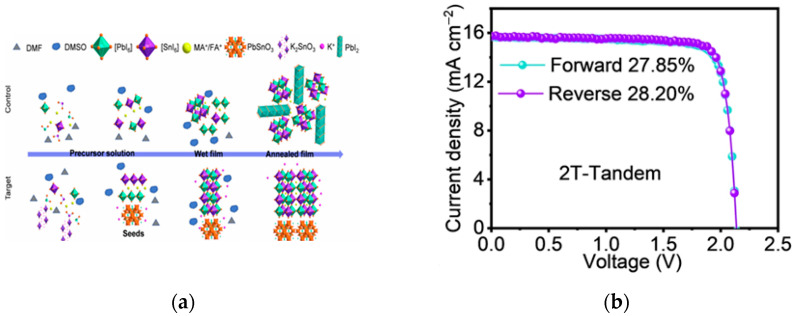

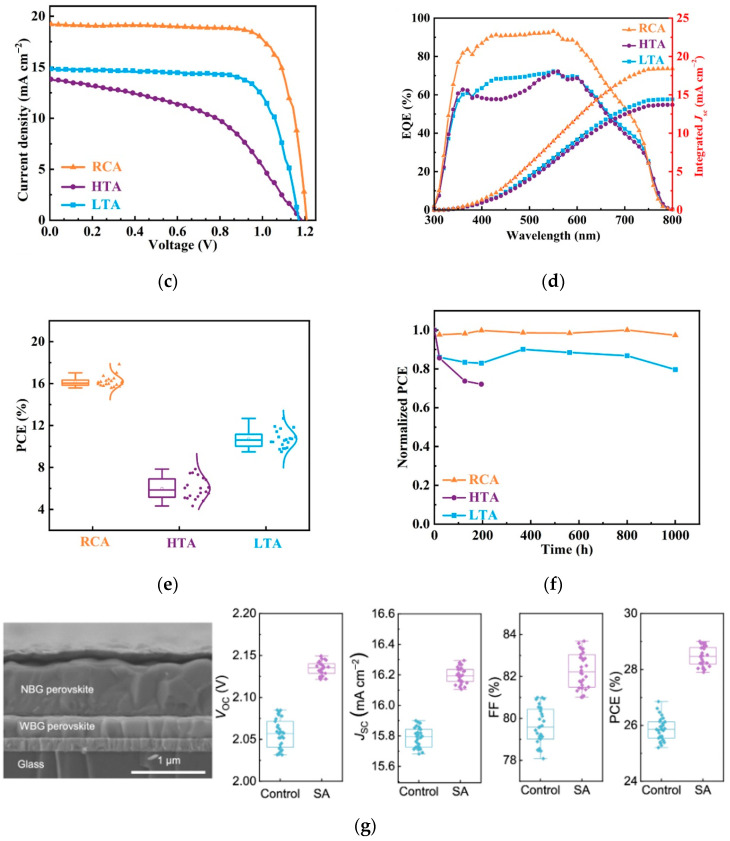
Research progress of all-perovskite tandem solar cells. (**a**) Schematic diagram illustrating the seed-induced oriented crystallization of K2SnO3-treated FA_0.7_MA_0.3_Pb_0.5_Sn_0.5_I_3_ perovskite films; (**b**) J-V curves of the champion 2T tandem PSCs [26] Copyright 2025, Nature Communication; (**c**) J-V curves; (**d**) EQE spectra, and (**e**) PCE statistics of PSCs based on RCA, HTA, and LTA; (**f**) normalized PCE evolution of PSCs without encapsulation stored in a nitrogen-filled glove box [27] Copyright 2024, Ecomat; (**g**) cross-sectional SEM image and photovoltaic performance statistics of SA-based all-perovskite tandem solar cells [28] 2025, Nature Communication.

Chen et al. adopted a seed-induced crystallization technique [26], introducing potassium stannate into the perovskite precursor to improve the quality of narrow-bandgap tin–lead mixed perovskite films. The efficiency of the all-perovskite tandem devices reached 28.12% (two-terminal) and 28.81% (four-terminal). The schematic diagram of seed-induced oriented crystallization of perovskite films and the corresponding device performance graphs are shown in Figure 5a,b. Xie et al. utilized a ligand evolution strategy with p-toluenesulfonyl hydrazide to regulate the nucleation and crystallization of all-inorganic narrow-bandgap perovskite films [27], successfully fabricating the first two-terminal all-inorganic perovskite tandem cell worldwide. It performed well in the 85 °C photothermal stability aging test, with a PCE of 21.92%; the device performance graphs are shown in Figure 5c–f. The interface compatibility between different layers in tandem cells is crucial for device performance. The team of Shi proposed a co-adsorption strategy using 6-aminohexane-1-sulfonic acid (SA)/[4-(3,6-dimethyl-9H-carbazol-9-yl)butyl] phosphonic acid (Me-4PACz) mixed SAMs to improve the quality of the wide-bandgap buried interface and reduce device V_oc_ loss [28]. The all-perovskite tandem device prepared based on this strategy achieved a PCE of 28.78%. The cross-sectional SEM image and photovoltaic performance graphs are shown in Figure 5g.

## 3. Strategies for Performance Improvement of PSCs

This section may be divided by subheadings. It should provide a concise and precise description of the experimental results, their interpretation, as well as the experimental conclusions that can be drawn.

### 3.1. Regulation of Element Composition

#### 3.1.1. Regulation of A-Site Ions

**Figure 6:**
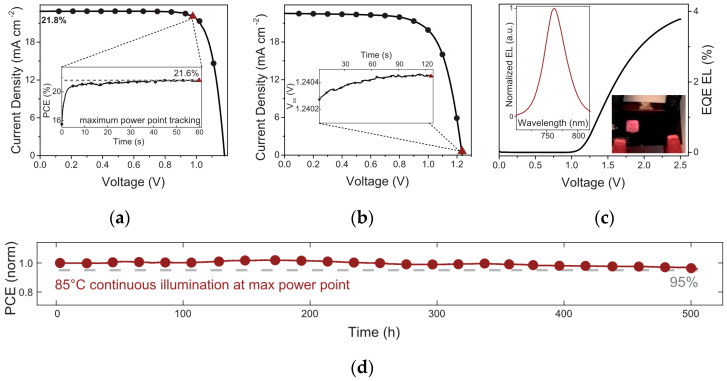

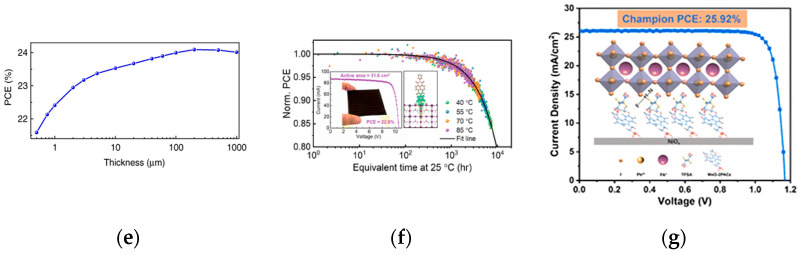
Photovoltaic performance of solar cells obtained via A-site ion regulation. (**a**) J-V curve measured at the maximum cell efficiency of 21.8%; (**b**) J-V curve measured at the maximum open-circuit voltage of 1.24 V; (**c**) external quantum efficiency and electroluminescence intensity as functions of voltage; (**d**) thermal stability test of the cell [30] Copyright 2016, Science; (**e**) PCE of the single crystal solar cells as a function of the thickness of the thin single crystals [31] Copyright 2017, Nature Communications; (**f**) performance of perovskite modules with DPNDI interface treatment [32] Copyright 2024, Energy and Environment Science; (**g**) efficiency of the micro-module based on TFSA-treated inverted PSC devices [33] Copyright 2024, Energy and Environment Science.

A-site cations are typically large organic or inorganic cations, such as MA^+^, FA^+^, and Cs^+^. They fill the gaps in the crystal structure, stabilizing the lattice structure and adjusting the crystal symmetry and lattice parameters. The radius and chemical properties of different cations affect the crystal structure and performance of perovskites. Mixed cation systems such as FA^+^/Cs^+^ mixtures can improve the crystallinity, stability, and photoelectric performance of PSCs. Studies by Yang have shown that an appropriate ratio of FA^+^/Cs^+^ mixture can fabricate efficient and stable PSCs [29]. FA^+^ helps form large-grain perovskite films, enhancing light absorption, but has the problem of high-temperature phase instability; Cs^+^ can enhance thermal stability and optimize phase stability and crystal structure. Saliba significantly improved the photostability and phase transition stability of perovskite materials by introducing double-cation (FA/MA) mixed perovskite materials [30], thereby promoting further efficiency improvement; the photovoltaic performance of solar cells obtained via A-site ion regulation is shown in Figure 6a–d. Chen et al. partially replaced MA with FA as the light-absorbing layer [31], obtaining a higher short-circuit current density (J_sc_). Thus, the device performance was improved, and the relevant results are shown in Figure 6e. The application of FA/MA mixed perovskites became an important breakthrough in perovskite materials at that time, making the PCE exceed 20% for the first time.

A-site ion regulation focuses on cations such as MA^+^, FA^+^, and Cs^+^, improving crystallinity, stability, and photoelectric performance through mixed systems. Recent studies have improved V_oc_ and J_sc_ through interface modification, with device efficiency reaching up to 25.92%, and significantly improved module stability. Tian et al. developed a naphthalene diimide derivative (DPNDI) [32], which forms strong coordination bonds with Pb^2+^ ions on the perovskite surface through pyridine groups, constructing a three-dimensional network Pb(II) coordination polymer layer at the interface. Perovskite modules treated with a DPNDI interface showed breakthrough performance, achieving a module efficiency of 22.6% and a 31.6 cm^2^ area. After 2000 h of continuous illumination at 85 °C, the module efficiency retained 85%; the relevant results are shown in Figure 6f. The T_80_ lifetime reached 9556 h, setting a global record for modules of the same size. The team of Chen developed tetrafluorosuccinic acid (TFSA) [33], which passivates uncoordinated lead defects through Lewis acid-base coordination, reducing the deep-level trap density. The inverted PSC device micro-module treated with TFSA achieved an efficiency of 22.78%, retained 84% of its efficiency after 600 h of thermal aging at 85 °C, achieving a key breakthrough in the perovskite field; the relevant results are shown in Figure 6g.

#### 3.1.2. Regulation of B-Site Ions

B-site ions are mostly transition metal cations (such as Pb^2+^, Sn^2+^, etc.), which form the core of the perovskite framework, form coordination bonds with X-site ions, and determine the photoelectric properties of the material, such as bandgap and carrier mobility. Meanwhile, in the selection of B-site ions, various elements have their own advantages. Although current research on lead-based perovskite materials is the most advanced, and the developed PSCs have absolute advantages in PCE, the inherent high toxicity of lead ions has always been a drawback. Therefore, the development of lead-free perovskite materials has become inevitable. To replace Pb in halide perovskites, researchers have selected several low-toxicity cations in the same period that are closest to Pb, such as Sn, Bi, Ge, etc., because they have similar inactive outer shell s orbitals, which are key to the unique photoelectric properties of perovskite materials.

Among these low-toxic or non-toxic materials, tin-based perovskites have high light absorption coefficients and narrow optical bandgaps, and tin halide perovskites have low exciton binding energy and good charge carrier mobility. Due to their similar ionic radius and valence state, tin (Sn) can partially or completely replace toxic lead (Pb) to achieve low-lead or non-toxic lead halide perovskites. However, divalent Sn ions are easily oxidized to tetravalent Sn ions, forming a self-doping process, resulting in poor device performance. To this end, various methods have been explored to enhance the stability of Sn-based optoelectronic devices.

**Figure 7:**
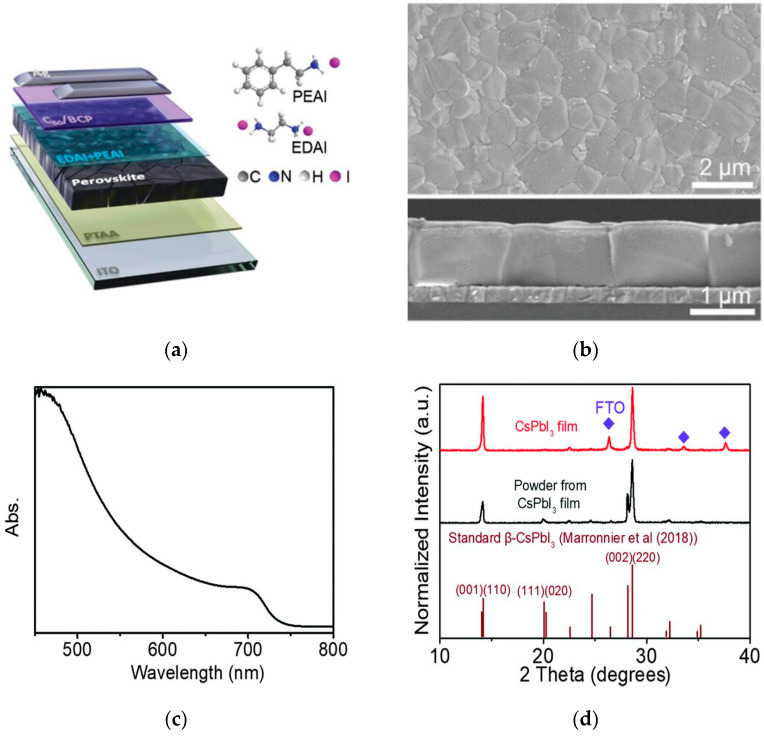
Characterization analysis of perovskite materials. (**a**) Diagram of device structure; the inset shows the chemical structure of the anchor molecules. (**b**) Surface-view and cross-sectional SEM images after STA treatment [34] Copyright 2022, Advanced Materials. (**c**) UV-Vis absorption spectrum. (**d**) XRD pattern [35] Copyright 2019, Science.

Pan et al. used 2-phenylethylamine hydroiodide (PEAI) and ethylenediamine hydroiodide (EDAI) as co-modifiers for surface treatment of perovskites [34], selectively anchoring active sites related to Pb and Sn and passivating defects of both metals. The device structure is shown in Figure 7a,b. Finally, V_oc_ of the lead–tin mixed perovskite solar cell was significantly increased from 0.79 V to 0.90 V, the V_oc_ loss was reduced to 0.43 V, and the device retained 80% of its initial efficiency after 2700 h of storage in nitrogen. Zhao et al. focused on all-inorganic tin-containing PSCs [35]. Considering their low toxicity, narrowest bandgap, and excellent thermal stability, they show great potential in single-junction and tandem PSCs. The device performance is shown in Figure 7c,d. However, problems such as the oxidation sensitivity of divalent Sn ions and the difficulty in regulating crystallization kinetics limit their development. This review analyzes their degradation mechanisms and discusses methods to improve efficiency and stability.

**Figure 8:**
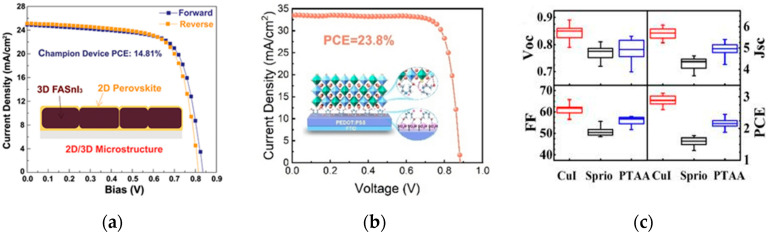
(**a**) J-V Curves of 2D/3D Heterostructured PSCs [37] Copyright 2021, Advanced Materials. (**b**) Structure of the molecules of the hole-modified layer and PEDOT:PSS films after hydroxylamine salt EOAI and DOAI treatment [38] Copyright 2024, Angewandte Chemie International Edition. (**c**) Statistics of photovoltaic parameters distribution of 20 Cs_3_Bi_2_I_9_ solar cells based on CuI, spiro-OMeTAD and PTAA [39] Copyright 2018, Elsevier B.V.

Theoretical calculations have shown that tin-based perovskites exhibit excellent optoelectronic properties (such as high carrier mobility, strong light absorption, and a direct bandgap), making them suitable for solar cells and other photovoltaic devices. However, the efficiency and stability of tin-based perovskite solar cell devices are still inferior to those of lead-based PSCs, mainly due to the instability of Sn^2+^ and the poor quality of tin-based perovskite films [36]. He et al. used fluorinated phenethylammonium bromide instead of formamidinium iodide (FAI) to prepare tin-based perovskite films [37]. This unique microstructure can inhibit the oxidation of some divalent Sn^2+^, ultimately achieving an efficiency of 14.81%. The device performance is shown in Figure 8a. Sun et al. developed multifunctional hydroxylamine salts EOAI and DOAI to modify the buried interface of Sn-Pb perovskites [38]. The treated PEDOT:PSS films are called PEDOT:PSS/EOAI and PEDOT:PSS/DOAI, respectively, and their structures, as well as the diagram of hydroxylamine salt action, are shown in Figure 8b. The optimized device has a fill factor of 81% and a PCE of 23.8%, retaining 95% of its efficiency after 2000 h of storage in nitrogen, providing a stable underlying foundation for all-perovskite tandem cells.

In addition, Bi is a non-toxic element and another candidate to solve the toxicity problem of lead-based perovskite materials. Bi has a similar electronic configuration, electronegativity, and ionic radius to Pb, but exhibits non-toxicity and high chemical stability. The perovskite structure formed by Bi-based materials is not easily deformed, and the introduction of halogen elements can transform the perovskite structure from tetragonal to cubic, further enhancing the stability of bismuth-based perovskite materials. Bai et al. fabricated high-quality lead-free Cs_3_Bi_2_I_9_ perovskite nanosheets film and further explore its potential application in solar cells with three different kinds of hole transport materials [39]. The solar cells based on ultrathin Cs_3_Bi_2_I_9_ nanosheets show remarkable improvement in the photovoltaic performance, and a high power conversion efficiency of 3.20% has been achieved in the solar cell with CuI as the hole transport material, which is the highest efficiency value of Bi-based perovskite solar cells that has been reported so far. The Cs_3_Bi_2_I_9_ nanosheets-based solar cell with CuI also showed a long-term stability in ambient air. The device performance is shown in Figure 8c.

B-site ion regulation mainly focuses on the development of low-toxic alternative materials such as Sn and Bi. Tin-based and Bi-based materials show great potential in single-junction/tandem cells due to their similarity to lead and low toxicity, but further solutions to Sn^2+^ oxidation and crystallization kinetics regulation are needed; bismuth-based perovskites have significantly improved performance by optimizing structural stability through Br doping. In the future, through continuous optimization of material stability and efficiency, lead-free perovskites are expected to achieve large-scale applications in the fields of photovoltaics and optoelectronic devices.

#### 3.1.3. Regulation of X-Site Ions

**Figure 9:**
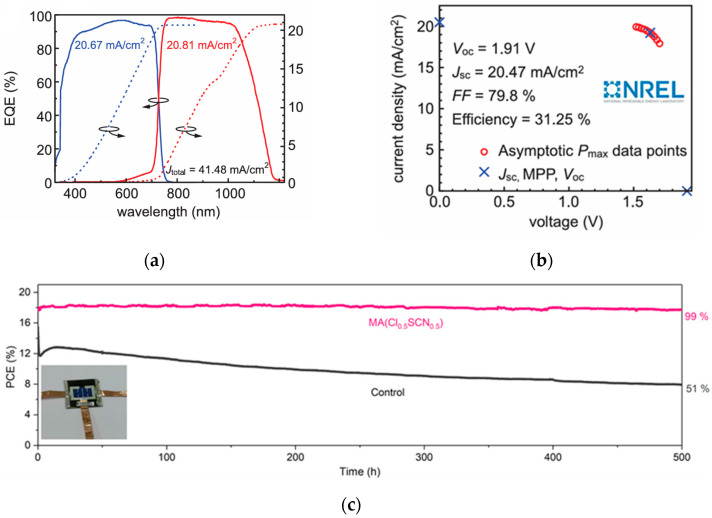

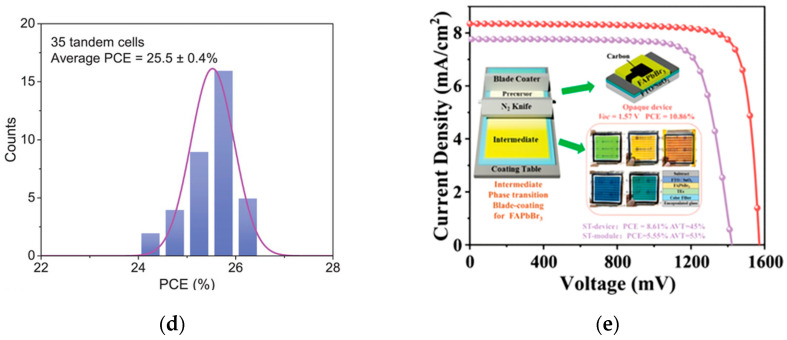
(**a**) Independently certified EQE; (**b**) asymptotic maximum power scan (right) [40] Copyright 2023, Science; (**c**) long-term stability of PSCs with additives [41] Copyright 2023, Adv. Mater; (**d**) histogram of PCEs over 35 tandem devices [42] Copyright 2022, Advanced Materials; (**e**) schematic of phase transition process from precursor to perovskite film and device performance [43] Copyright 2023, Adv. Mater.

X-site ions are generally halogen anions (such as I^−^, Br^−^, Cl^−^, etc.), which form an octahedral coordination structure with B-site ions. The selection of different X-site ions can adjust the bandgap and photoelectric performance of perovskites. For example, I^−^ can narrow the perovskite bandgap, facilitating the absorption of long-wavelength light, but this easily leads to phase instability. Br^−^ can increase the bandgap and improve the open-circuit voltage. Tan et al. developed a tandem structure with Cs_0.18_FA_0.82_Br_3_ as the top cell [40,41], adjusting the bandgap to 1.67 eV through Br^−^ doping, and combining interface optimization, ultimately achieving a PCE of 31.25%. The device performance is shown in Figure 9a–c. Introducing a small amount of Cl^−^ into perovskites can improve crystallization quality, reduce defect state density, and enhance device efficiency and stability. Wen et al. introduced Cl^−^ into wide-bandgap perovskites [42], while reducing lattice strain and non-radiative recombination. The optimized device has an open-circuit V_oc_ of 1.26 V and a PCE of 17.7%, retaining over 90% efficiency after 1000 h of long-term operation. The device performance is shown in Figure 9d. By mixing different X-site ions (such as I^−^/Br^−^, I^−^/Cl^−^), precise bandgap adjustment can be achieved, broadening the light absorption range and optimizing device performance. Yue et al. developed a strategy to induce the formation of intermediate phases via precursor solvents [43], thereby facilitating the fabrication of high-quality FAPbBr_3_ films. When carbon was employed as the top electrode, the device achieved an efficiency exceeding 10%. Moreover, it retained 96% of its power output after 500 h of maximum power point tracking tests, with an estimated lifespan of up to 2500 h. The device performance is shown in Figure 9e.

The unique ABX_3_ structure of perovskites enables the combination of different elements, providing directions for improving performance and practicality. A-site mixed cations and interface modification technologies have driven breakthroughs in device efficiency and stability, paving the way for large-area module applications. Lead-free development at the B-site is a core direction; tin-based and bismuth-based materials need to address issues such as Sn^2+^ oxidation and crystallization kinetics. The mixed system of X-site halogen ions provides flexible solutions for precise bandgap regulation, thereby broadening the light absorption range and reducing voltage loss. In the future, through multi-dimensional synergistic regulation of ions, perovskite materials are expected to achieve efficient, stable, and non-toxic large-scale applications in the fields of photovoltaics and optoelectronic devices.

### 3.2. Regulation of Perovskite Charge Transport Layers

In PSCs, charge transport layers (including ETLs and HTLs) are core components, and their performance directly affects the power conversion efficiency, stability, and charge separation/transport efficiency of the cells. The interfaces between the perovskite layer and ETLs/HTLs are key regions for charge separation and transport. Interface defects or energy level mismatches can lead to severe charge recombination, thereby reducing cell performance. The defect issues at these interfaces can be addressed through passivation. Modifying the surface with insulating layers or organic molecules can seal oxygen vacancies and block ion migration toward ETLs. Additionally, the passivation effect of dipolar molecules can be used to inhibit interfacial non-radiative recombination, and the use of multifunctional hydroxylamine salts to modify the buried interface can effectively reduce interface defects, decrease the degree of non-radiative recombination, and optimize device stability. For ETLs, surface modification can be performed to enhance their interaction with the perovskite layer. For HTLs, on one hand, new materials are developed, and hole transport materials (HTM) with high mobility, good energy level matching, and stability are selected. On the other hand, self-assembled monolayer (SAM) materials are used to modify the interface between HTLs and perovskite to regulate molecular orientation. These regulatory measures can improve the efficiency of charge extraction and transport, reduce recombination at the interfaces, and thus enhance the efficiency and stability of the cells.

#### 3.2.1. ETL Interface Modification

**Figure 10:**
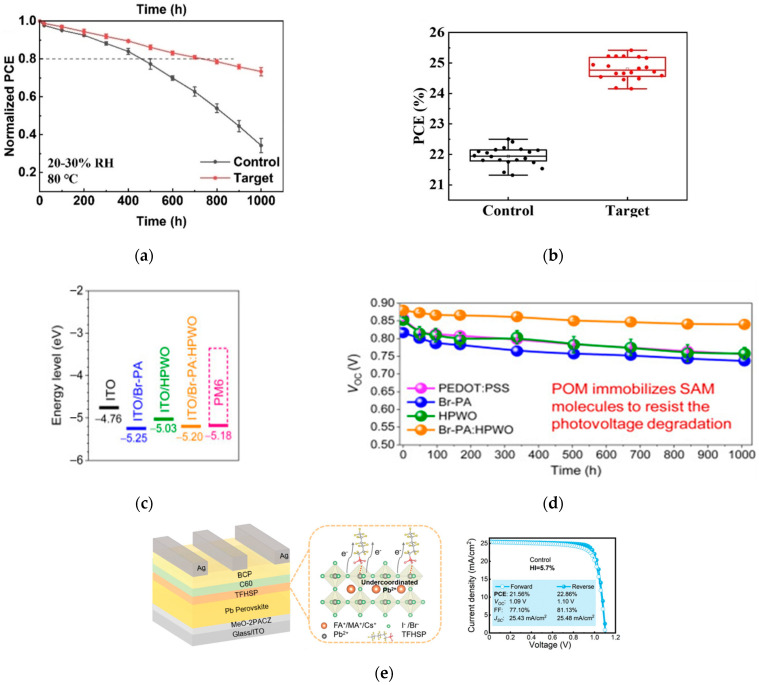
(**a**) Thermal stability tests of unencapsulated control and target devices at 80 °C and 20–30% RH. (**b**) Statistical photovoltaic performance of control and target devices [44] Copyright 2025, Angewandte Chemie International edition. (**c**,**d**) Stability of devices under 65 °C thermal aging [45] Copyright 2024, Joule. (**e**) Schematic of device structure, interaction between TFHSP and perovskite, and J-V curve of champion PSC with TFHSP [46] Copyright 2024, Advanced Materials.

Song introduced 2,2′-bipyridine-4,4′-dicarboxylic acid (HBPDC) at the interface between the SnO_2_ electron transport layer and perovskite [44]. HBPDC can not only reduce the surface defects of SnO_2_ but also passivate the perovskite defects. This dual-functional modification optimizes the interface energy level matching and promotes charge transport, increasing the device efficiency to 22.6%; the device performance is shown in Figure 10a. Bao et al. proposed a composite HTL of SAM and polyoxometalate [45], optimizing the interface through multiple p-type doping. The organic solar cell based on this composite HTL achieved an efficiency of 19.1%; the device performance is shown in Figure 10b. The research group of Wu introduced potassium tridecafluorohexane-1-sulfonate as an intermediate layer [46], which significantly reduced the surface and interface defect density, optimized the energy band arrangement, and reduced charge accumulation. The efficiency of the optimized device reached 24.6%; the device performance is shown in Figure 10c,d.

#### 3.2.2. HTL Interface Modification

**Figure 11:**
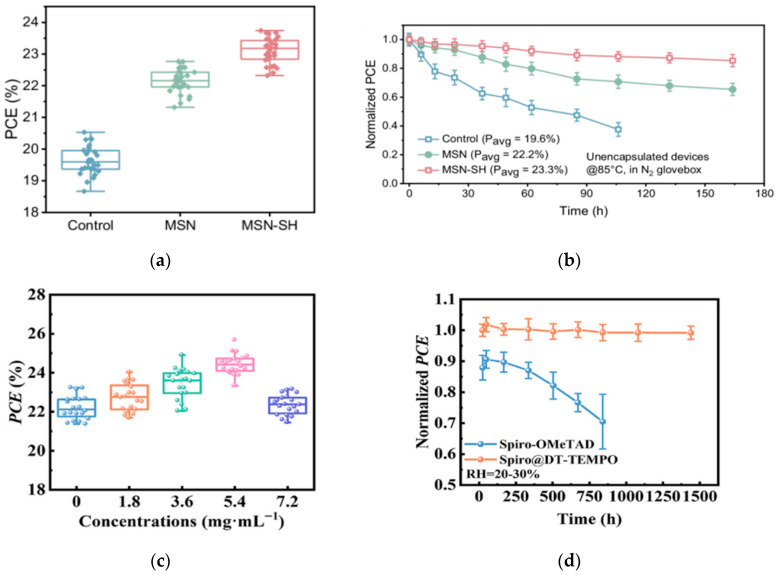
(**a**) Statistical distribution of PCE for control, MSN, and MSN-SH Sn-Pb PSCs based on 30 devices. (**b**) Performance evolution of unencapsulated control, MSN, and MSN-SH Sn-Pb PSCs aged at 85 °C under dark conditions in the N_2_ glovebox [47] Copyright 2025, Nature Communications. (**c**) The PCE histograms were obtained from 20 PSCs doped with different concentrations of DT-TEMPO. (**d**) Stability of unencapsulated devices in air with error bars indicating the standard deviations from 10 samples [48] Copyright 2025, Advanced Materials.

The team of Wang developed a strategy for reconstructing the buried interface of Sn-Pb perovskite films [47]. They used mercapto-functionalized mesoporous silica nanoparticles (MSN-SH) as the upper scaffold on the acidic PEDOT:PSS substrate, which improved the mechanical toughness of the PEDOT:PSS/perovskite interface. Combined with the optimized wide- and narrow-bandgap perovskite sub-cells, they fabricated a double-junction all-perovskite tandem device with a maximum efficiency of 29.63%. The device also exhibited enhanced light and thermal stability, retaining approximately 90% of its initial efficiency after 445 h of continuous operation. The statistical distribution of PCE and performance evolution of unencapsulated control are shown in Figure 11a,b. Yang et al. introduced a multifunctional self-healing nitroxide radical monomer DT-TEMPO [48]. Through p-type doping, it improved the hole mobility and conductivity of Spiro-OMeTAD, while optimizing the energy level alignment and charge transfer process between the perovskite and Spiro-OMeTAD. The optimized perovskite solar cell achieved a certified photovoltaic conversion efficiency of 25.30% on a rigid substrate. The device performance is shown in Figure 11c,d.

The regulation of charge transport layers has effectively optimized the energy level matching and charge transport efficiency between ETL/HTL and perovskite through surface modification, new material development, and SAM interface engineering. Through strategies such as organic molecules, dipolar molecules, hydroxylamine salts, and chelating ligands, interface defects and non-radiative recombination have been significantly reduced, and the efficiency and long-term stability of devices have been improved. These studies have laid a solid foundation for the large-scale application of PSCs.

### 3.3. Research on Stability

The long-term stability of PSCs is a core obstacle to their commercialization. To address decomposition and performance degradation caused by external factors such as humidity, thermal stress, ultraviolet light, and electric fields, current solutions mainly include material composition engineering, interface modification, high-efficiency encapsulation, and device structure innovation.

**Figure 12:**
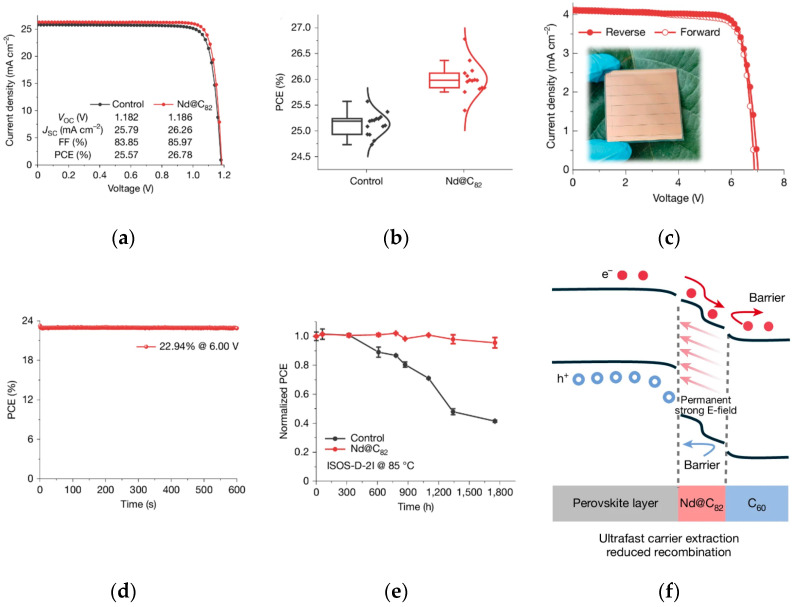
Photovoltaics and stability of Nd@C_82_-based PSCs and modules. (**a**) J–V curves of the champion control and Nd@C_82_-based PSCs under 1-sun (100 mW cm^−2^) illumination. (**b**) The statistics of PCE values obtained from J–V characteristics for the control and Nd@C_82_-based devices. (**c**) J–V curves of champion modules (25 cm^2^) based on Nd@C_82_. (**d**) The stabilized PCE of champion modules based on Nd@C_82_. (**e**) ISOS-D-2I device stability during storage at 85 °C for 1750 h (where ISOS is the International Summit on Organic Photovoltaic Stability, five devices for each type). The error bars denote standard deviation. In cases where error bars are not visible, they are smaller than the symbol size. (**f**) Energy diagram for Nd@C82-based PSCs [50] Copyright 2025, Nature.

By regulating the chemical composition of perovskite materials, the content of easily decomposable components (e.g., MA^+^) and ion migration are reduced, thereby inherently enhancing resistance to external interference. The research group of Lin significantly reduced film pores and enhanced light absorption by doping N719 dye molecules into the perovskite precursor [49]. The hydrophobic properties and hydrogen-bonding interactions of N719 effectively delayed the hydration decomposition of perovskites, providing a new strategy for humidity-sensitive devices. The team of Liang pioneered a coupled structure of endohedral metallofullerene (Nd@C_82_) and polymethyl methacrylate [50], which inhibits ion migration through strong interfacial polarization effects. In the ISOS-D-3 standard damp-heat test, the device retained 99% of its efficiency after 1000 h of operation, demonstrating excellent water vapor barrier capability. The photovoltaic performance and stability of Nd@C_82_-based PSCs and modules are shown in Figure 12.

**Figure 13:**
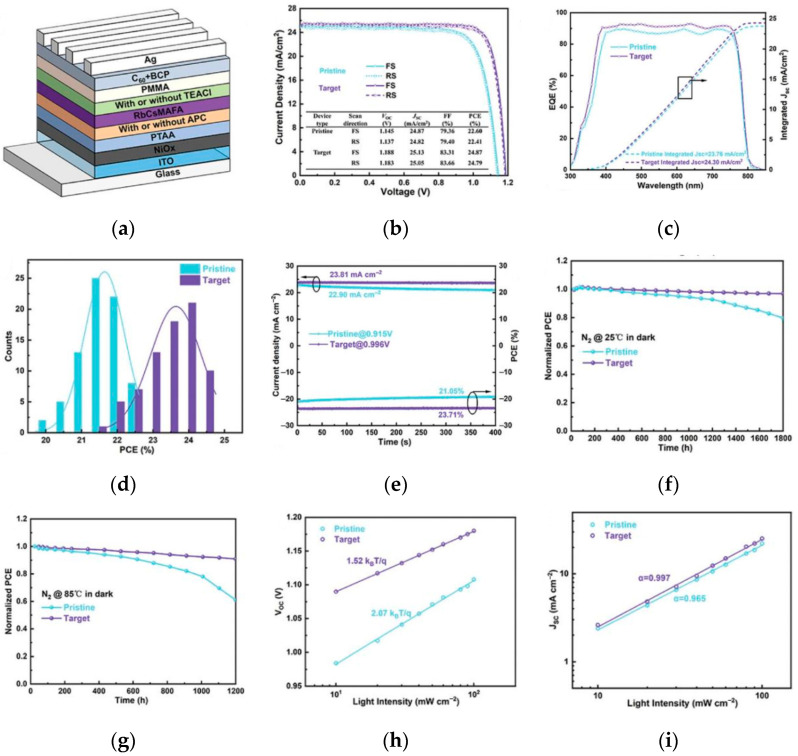
(**a**) Device structure. (**b**) J–V curves of the best-performing devices for pristine and target conditions. Inset shows the detailed parameters of PSCs. (**c**) EQE spectra and the corresponding integrated JSC. (**d**) Histograms of PCE distribution among 75 devices. (**e**) Steady-state photocurrent and output PCE at the maximum power point; long-term stability test for the unencapsulated corresponding devices under. (**f**) 25 °C for 1800 h and (**g**) thermal stress of 85 °C condition. (**h**,**i**) V_oc_ and J_sc_ versus light intensity of the corresponding devices [51] Copyright 2024, Advanced Functional Materials.

By modifying the interfaces of the PSCs, where the perovskite contacts with the electron transport layer (ETL) and hole transport layer (HTL), defects are reduced to decrease the probability of carrier complexation and ion diffusion, thus enhancing the stability. Xu et al. used 5-aminopyridine-2-carboxylic acid to modify the buried bottom interface [51], 2-thiophene ethylamine chloride to passivate the top interface to significantly inhibit ion migration. The unencapsulated device retains 90% of the initial efficiency after aging at 85 °C for 1200 h, and the activation energy for ion migration increases to 252 meV. The device structure and performance are shown in Figure 13.

**Figure 14:**
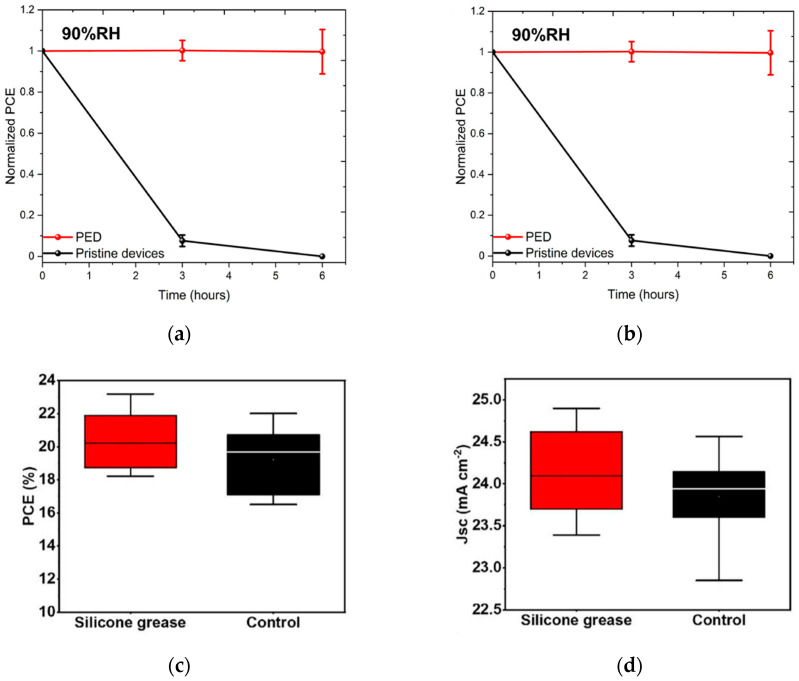

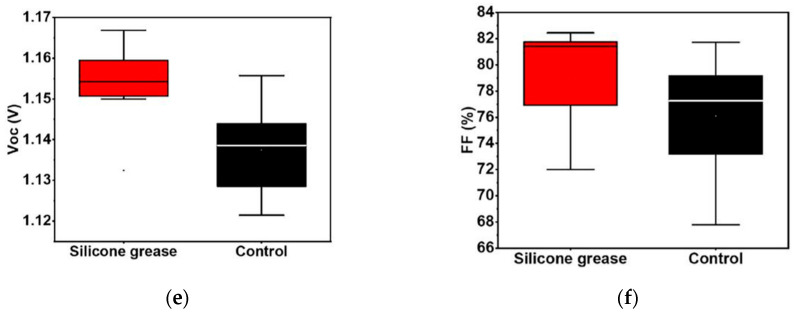
(**a**) Normalized PCE vs. time graphs of the 90% RH test. (**b**) The J-V behavior of PED and pristine champion devices before and after 24 h of rest outdoors at −16 °C [52] Copyright 2024, Materials Today Energy. Statistics of (**c**) PCE, (**d**) V_oc_, (**e**) J_sc_, and (**f**) FF of PSCs based on vacuum silicone grease packaging [53] Copyright 2025, Advanced Functional Materials.

A physical barrier can be established between PSCs and the external environment by modifying encapsulation materials, selecting appropriate encapsulation methods, and improving encapsulation technologies to prevent rapid degradation of PSCs in harsh environments. Mousavi et al. developed a polydimethylsiloxane (PDMS) encapsulation technology [52], where PDMS is directly coated on the surface of PSCs through a one-step encapsulation process. The PDMS coating not only reduced the reflectivity in the visible light region by 50% and increased PCE by 8%, but also extended the stability of the encapsulated device to 6 h under 90% humidity, with 80% efficiency retained after 360 h of storage, achieving both optical optimization and environmental barrier. The fabrication process of AR-PED is shown in Figure 14. The team of Li developed a vacuum silicone grease encapsulation technology [53], using PDMS-based silicone grease as the core material. The grease is coated on the surface of PSCs at low temperature and forms a seamless barrier through vacuum lamination. Due to defect inhibition, which reduces non-radiative recombination, the PCE increased from 23.91% to 25.34%. Under 90% humidity, the encapsulated device retained 95% of its initial efficiency after 1000 h, achieving a dual breakthrough in efficiency and stability.

**Figure 15:**
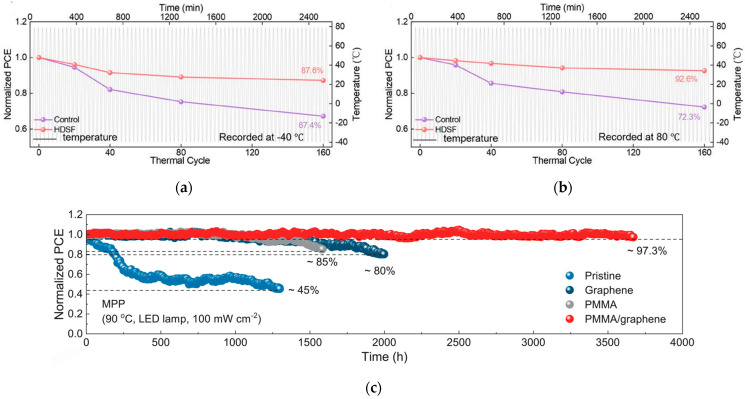
(**a**) −40 °C and (**b**) −80 °C of control and HDSF-treated devices against thermal cycles between −40 °C and 80 °C [54] Copyright 2025, Advanced Materials. (**c**) Under standard solar illumination and high-temperature conditions, the solar cell achieves a T97 operational lifetime of 3670 h [55] Copyright 2025, Science.

The tolerance of PSCs to complex environments can be improved by optimizing the device structure or introducing special mechanisms. Due to its unique pin structure, the inverted structure allows ETL to block direct contact between external water/oxygen and perovskite, while HTL is closer to the substrate, reducing interface peeling under thermal stress; the all-inorganic structure replaces organic transport layers with inorganic HTL and ETL, avoiding thermal oxidation or hydrolysis of organic materials. The team of Li designed a functional molecular framework containing disulfide bonds [54], which can real-time repair perovskite lattice damage through dynamic bond exchange during temperature fluctuations. After 160 cycles from −40 °C to 80 °C, the optimized device retained 87.6% and 92.6% of its initial efficiency, respectively, with a certified efficiency of 25.84%. The device performance is shown in Figure 15a,b. The team of Hou enhanced the perovskite lattice through the use of PMMA-coupled single-layer graphene [55], thereby inhibiting photo-mechanically induced decomposition. The device achieved a T97 lifetime of 3670 h under 90 °C standard illumination, setting a new record for high-temperature stability. The statistics of device performance are shown in Figure 15c. Future research efforts should focus on molecular design, with an emphasis on improving interface stability and reducing defect states to further enhance the V_oc_ and J_sc_ of perovskite devices.

## 4. Machine Learning Applications in PSCs

PSCs have garnered significant research interest due to their excellent photovoltaic properties, such as tunable bandgaps and high carrier mobility. However, traditional perovskite material development and device fabrication processes are heavily reliant on time-consuming and labor-intensive trial-and-error experimentation. As a data-driven fourth paradigm, ML addresses this challenge by abstracting real-world problems into mathematical models solved via computational power. This approach offers a viable solution to managing complex research data in the PCS field. The 2011 US Materials Genome Initiative championed a “trinity” approach integrating computation, data, and experiment to supplant the traditional trial-and-error model of materials research and development. This initiative has spurred the emergence of research that integrates perovskite solar cells with machine learning, which showcases significant potential [56].

### 4.1. Open-Access Databases and Analysis Tools

Initially, ML mainly focused on predicting fundamental properties of perovskites, such as the bandgap and formation energy. In contrast, density functional theory, which is firmly grounded in quantum mechanics, provided greater accuracy. Nevertheless, ML established its own position in material property prediction because of its remarkably low computational cost. As research progressed, ML methods demonstrated considerable potential for screening the interface materials of PSC devices. However, the performance of ML is highly reliant on large quantities of high-quality data. As a result, data accumulation and model optimization emerged as the key directions for development.

**Figure 16:**
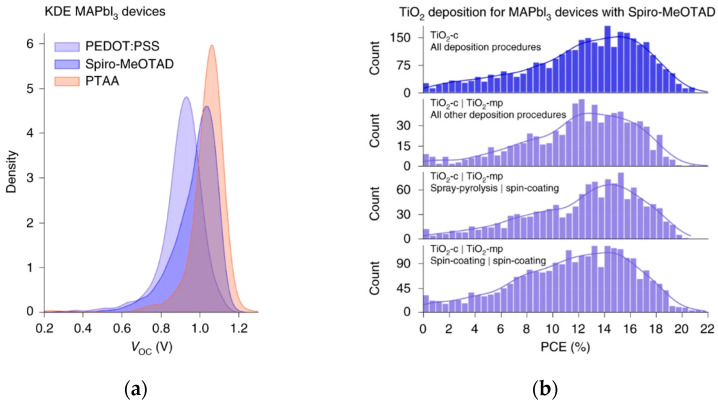

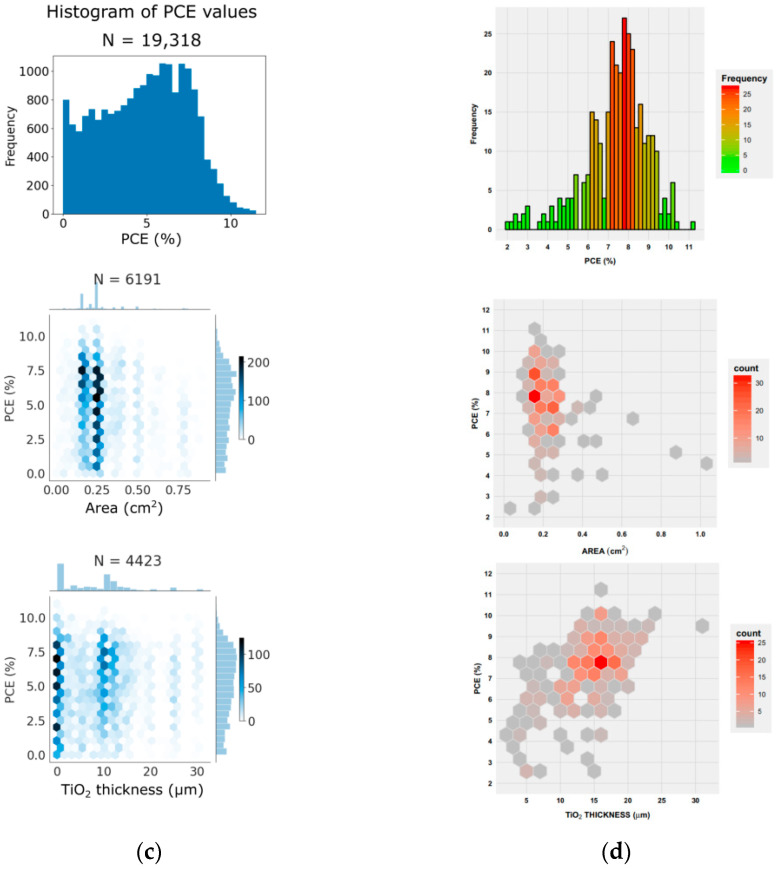
Example of analysis from the database. (**a**) The kernel density estimation of the V_oc_ for three common HTLs for MAPbI_3_-based devices. (**b**) Performance distributions separated by deposition procedures for the TiO_2_-ETL in nip devices with MAPbI_3_ and Spiro-MeOTAD [57] Copyright 2022, Nature Energy. (**c**) Graphs comparing the new automatically created DSC database to (**d**) the manually created database ‘DSSCDB’ [58] Copyright 2022, Scientific data.

Data sharing establishes critical infrastructure. Jacobsson et al. developed an open-access database and analysis tool for PSCs based on FAIR (Findable, Accessible, Interoperable, Reusable) data principles [57]; Figure 16b shows the deposition process distribution of TiO_2_-ETL, which provides a data basis for ML optimization of large area processes. The Perovskite Database project provides access to device data and visualization tools for interactive data exploration. By spring 2020, it had incorporated data from over 42,000 devices documented in peer-reviewed literature. Analysis using this database demonstrates capabilities for investigating tandem cell integration, stability, and scalability. This project optimizes data management and sharing culture in perovskite research, setting a benchmark for other experimental fields and accelerating further development of ML and other AI approaches.

Beard et al. constructed a database containing photovoltaic characterization and material data for PSCs [58]. Parsing 25,720 publications using ChemDataExtractor yielded 660,881 data points covering 57,678 photovoltaic devices. A hybrid manual–automated multidimensional validation ensured data quality, achieving accuracy metrics between 73.1% and 95.8%. This database facilitates data-driven research and development of photovoltaic materials, addressing the urgent demand for renewable energy technologies amid the climate crisis. It also provides a centralized platform for storing PV performance metrics relevant to third-generation PV devices.

In conclusion, open databases are foundational for deep ML integration. Early limitations in data volume constrained the accuracy of ML predictions, making data accumulation and model optimization crucial breakthroughs. Databases constructed following FAIR principles and via extensive literature parsing provide rich, high-quality data resources. These platforms significantly enhance data management and sharing, laying a robust foundation for the advancement and application of ML in perovskite solar cell research.

### 4.2. Data-Driven Material Screening

Among the numerous perovskite materials, identifying those that meet performance requirements is extremely challenging. However, ML, with its robust data processing and predictive capabilities, provides a powerful tool for screening PSC device materials. ML offers powerful capabilities for PSC material screening by processing large datasets and establishing predictive models. While ML cannot guarantee the identification of materials with specific properties, it efficiently narrows down the candidate pool. This capability stems from ML algorithms’ aptitude for learning complex patterns from data combined with appropriate model interpretation techniques. This data-driven paradigm revolutionizes the traditional trial-and-error experimental approach, substantially reducing experimental costs and enhancing the efficiency of research and development.

**Figure 17:**
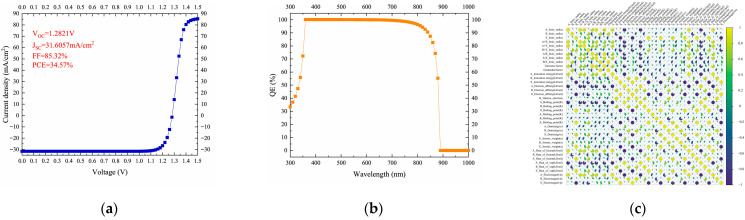

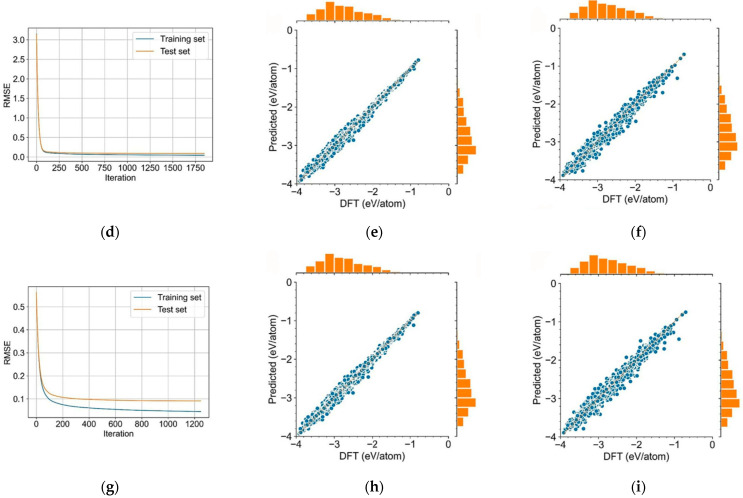
Optimized cell performance (**a**) QE, (**b**) J-V [59] Copyright 2024, Elsevier B.V.; (**c**) correlation heatmap of the perovskite dataset [60] Copyright 2024, Elsevier Inc.; Evaluation of ML models on formation enthalpy prediction; (**d**,**g**) loss function of XGBoost, LightGBM, and Random Forest algorithms on training and test datasets, respectively; (**e**,**f**) XGBoost predictions vs. DFT calculations on training and test sets; (**h**,**i**) LightGBM predictions vs. DFT calculations on training and test sets [61] Copyright 2025, Elseviers.

Lu et al. replaced the electron and hole transport layers of the perovskite cell with doped CsGeI_3_, and used SCAPS software to build various cell models and optimize the internal parameters of the cells [59]. As a result, PCE of the cells has surged from 17.91% to an impressive 34.57%, representing an enhancement in cell performance. Zhu et al. integrated density functional theory calculations with machine learning algorithms and established a comprehensive perovskite database [60]. The optimized cell performance are shown in Figure 17a,b. They trained and optimized ML models using 488 DFT-calculated datasets, then expanded the database to 177,264 datasets and conducted three-stage high-throughput screening based on stability, band gap, and photovoltaic performance. The correlation heatmap of the perovskite datase are shown in Figure 17c. The XGBoost algorithm was selected as the optimal model, and four lead-free perovskite solar cell materials with SLME values exceeding 23% were successfully predicted. These findings provide valuable insights for the development of stable, efficient, and green lead-free perovskite solar cells. Zhang et al. integrated ML and symbolic regression for screening multicomponent perovskite oxides [61]. XGBoost and LightGBM achieved high accuracy (R^2^ = 0.98) in predicting formation enthalpy for 6526 compositions, with oxidation state and electronegativity as input features. Symbolic regression addressed ML’s “black-box” issue by deriving an explicit functional relationship (R^2^ = 0.79), enhancing interpretability. Combined with high-throughput screening and DFT simulations, they identified CeEuAl_2_O_6_ as a prime photovoltaic candidate with a 2.31 eV direct bandgap, the Evaluation of ML models on formation enthalpy prediction are shown in Figure 17d–i.

**Figure 18:**
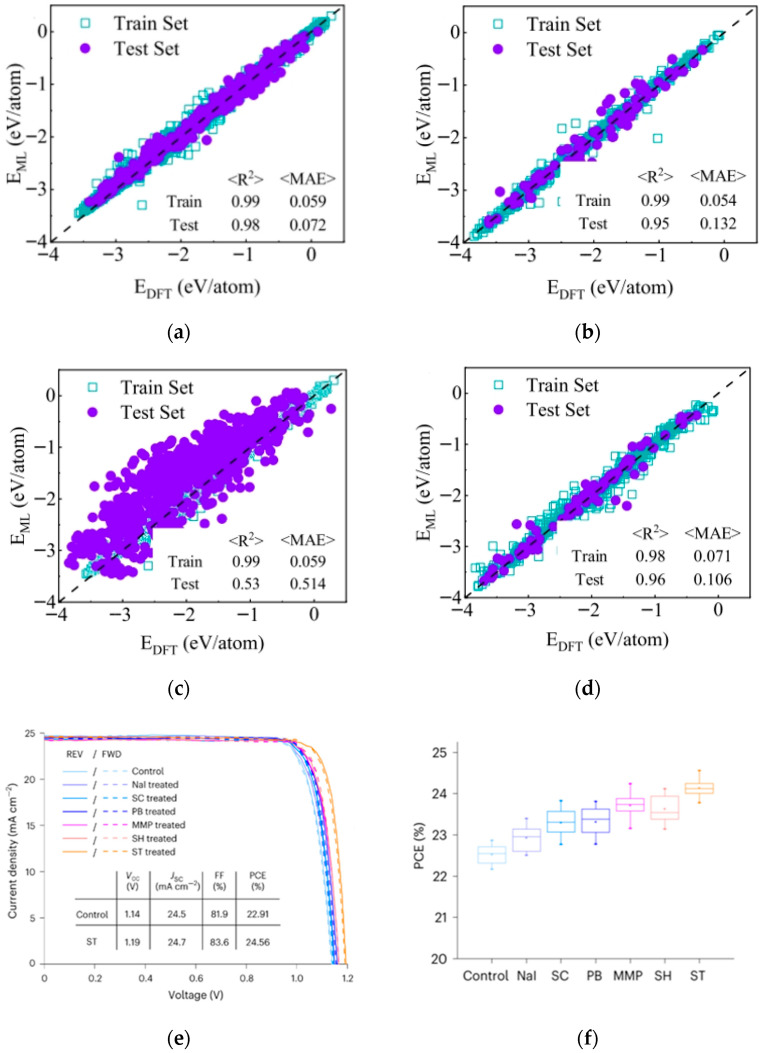
Formation energies predicted by machine learning-based models and DFT using various datasets (**a**) spinel oxides, (**b**) perovskite oxides, (**c**) direct prediction of perovskite oxides using the source domain model, (**d**) transfer learning model [62] Copyright 2023, NPJ Computational Materials. (**e**) J-V scans of the control, NaI, and five PH anion-treated devices. (**f**) Comparison of PV performance among control, NaI, and five PH anion-treated PSCs [63] Copyright 2023, Nature Materials.

Liu et al. tackled the challenge of small perovskite datasets for formation energy prediction using deep learning coupled with transfer learning [62]. They pre-trained the model using abundant data from spinel structures (lattice-similar to perovskite) and then fine-tuned it with limited perovskite data. This strategy achieved formation energy prediction accuracy exceeding that of models trained solely on the limited perovskite data, providing an effective solution for material property prediction with sparse data. The formation energies predicted by machine learning-based models and DFT using various datasets are shown in Figure 18a–d. Xu et al. focused on screening passivators for perovskite surfaces [63]. They developed an ML model based on physical descriptors, using DFT-calculated binding energies as training data. Analysis of factors like molecular oxygen atom count and topological polar surface area revealed correlations with binding energy. The model identified 24 potential pseudo-halide (PH) anions with binding energies > 3 eV, among which 15 exhibited dual passivation capabilities. Sodium thioglycolate emerged as the top performer, enabling efficient screening for perovskite surface defect passivation. The J-V scans and comparison of PV performance among control, NaI, and five PH anion-treated PSCs are shown in Figure 18e–f.

The data-driven paradigm transforms research and development methodology. In PSC research, ML-based data-driven screening significantly narrows the candidate material space by processing vast datasets and uncovering hidden correlations between material features and performance. This substantially reduces experimental costs and boosts efficiency. This innovation spans the entire development chain from theoretical screening to experimental validation, offering precise guidance and accelerating the discovery of high-performance PSC devices.

### 4.3. Accurate Prediction of Material Properties

**Figure 19:**
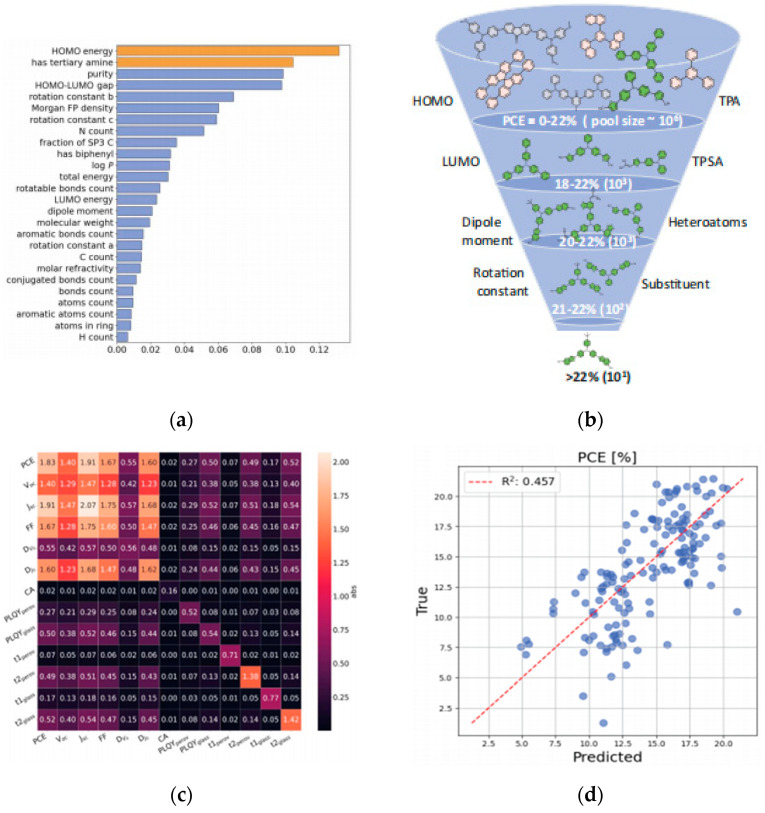
(**a**) Feature importance for predicting HTM performance via ML. (**b**) Hierarchical PCE contribution of HTM molecular features, with % ranges showing influence on PSC performance. (**c**) Heatmap of predicted vs. experimental PCE for HTM library, color-coding values to identify high-performance candidates. (**d**) Correlation of predicted (x) vs. true (y) PCE for HTMs in PSCs, with R = 0.457 quantifying model accuracy [64] Copyright 2024, Science.

**Figure 20:**
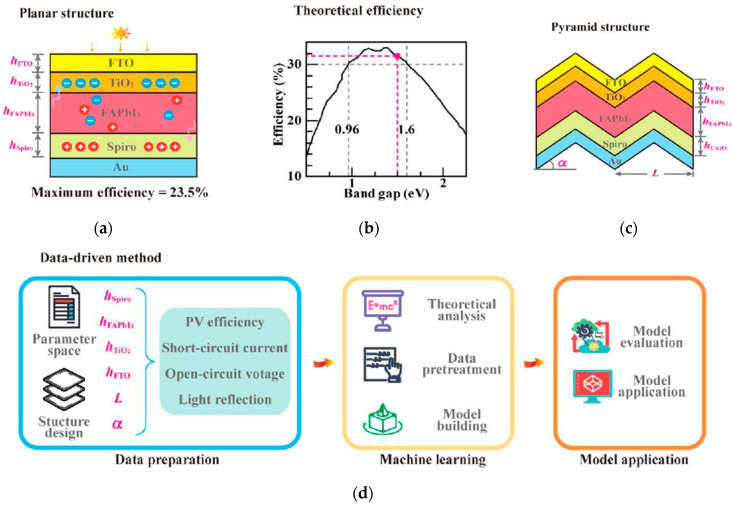
(**a**) Structure and parameters of planar PSC (hFTO, hTIO2, FAPbI3, and Spiro). (**b**) Relationship between the conversion PCE and bandgap, where the PCE is >30% when the bandgap is between 0.96 and 1.6 eV. The bandgap of FAPbI_3_ considered in this paper is 1.5 eV, and the corresponding theoretical PCE is 31.6%. (**c**) Structure and parameters of pyramid PSCs. The parameters L and α describe the period and tilt angle, respectively, of the pyramid structure compared to the planar structure. (**d**) Flowchart of neural network analysis of the structural parameters of PSCs, including data collection, network learning, and model application process [65] Copyright 2024, The Journal of Physical Chemistry Letters.

**Figure 21:**
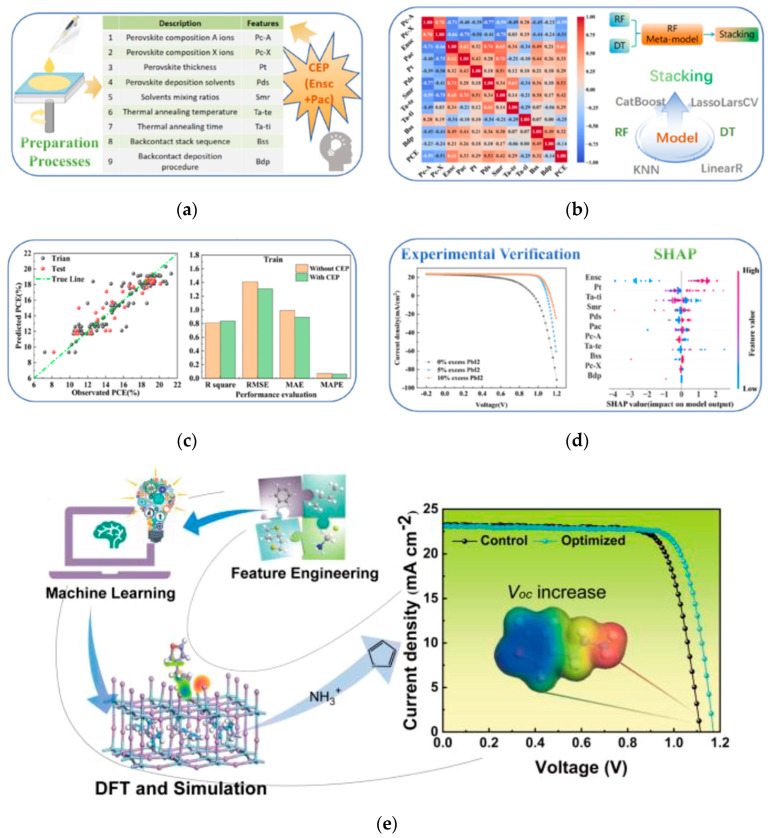
Machine learning workflow in this study. (**a**) Preparation processes and dataset collection (Ensc and Pac are two input features used as CEPs), (**b**) model building, (**c**) performance evaluation, and (**d**) SHAP interpretation and experimental verification APL materials. (**e**) Machine learning maps the relationship between interface material and device performance, aiming to intelligently screen materials to minimize voltage losses for efficient p-i-n type PSCs [66] Copyright 2023, Journal of Energy Chemistry.

The core value of ML in predicting perovskite properties lies in transforming the complex “feature-microstructure-macro property” relationship into a computable model, overcoming traditional experimental limitations. ML leverages decisive core features impacting perovskite performance:

1. Chemical composition and doping: Cation/anion combinations (e.g., mixed cation ratios, halide ratios) directly influence bandgap width and carrier mobility. Dopant type and concentration impact defect density and stability. Wu et al. employed Gaussian Process Regression as the surrogate model for Bayesian optimization to optimize the design of HTMs [64]; Figure 19d shows the scatter plot of HTM performance prediction with R^2^ = 0.457, showing the positive correlation between molecular weight and PCE, which provides a direction for the modification of Spiro-OMeTAD in Section 3.2.2. Through virtual screening and experimental validation, they significantly reduced the time consumption associated with traditional trial-and-error approaches. The study concludes by emphasizing the dual strategies derived from training the ML model, which is capable of predicting complex properties such as device performance based on molecular structure inputs.

2. Crystal structure and microstructure: Key morphological parameters are crucial for predicting stability and carrier transport. Zong et al. took single-junction FAPbI_3_ PSCs as the research object [65], combined neural network models with high-throughput simulation calculations, and compared the performance of planar and pyramid structures. The results, as shown in Figure 20, indicate that the pyramid structure, through the light-trapping effect, increases the Jsc to 27.4 mA/cm^2^ and the PCE to 28.4%, both exceeding the existing experimental results. The optimized periodicity and tilt angle of the pyramid match the textured structure of crystalline silicon solar cells. This study highlights the potential of neural network inverse design based on high-throughput calculations in the field of optoelectronic devices.

3. Fabrication process parameters: Solution processing, such as solvent polarity, anti-solvent dripping time, precursor concentration impact film uniformity. Thermal treatment, including annealing temperature, duration, and atmosphere, influences the crystallinity of perovskite films. Li et al. utilized a coarse estimation procedure (CEP) strategy to optimize an ML model analyzing the impact of 11 process parameters on PCE [66]. Analysis revealed significant interaction effects: antisolvent dripping time (20–30 s) combined with precursor concentration (1.2–1.5 mol/L) minimized surface roughness (<5 nm), increased crystallinity (~20%), and yielded devices with 23.1% PCE. Furthermore, annealing at 100–120 °C for 30 min effectively suppressed crystal defect formation, enhancing stability. The machine learning workflow in this study are shown in Figure 21.

4. Interface and device characterization: The matching of charge transport layer, energy levels of ETL/HTL, conductivity, energy level offsets at the ETL/perovskite and HTL/perovskite interfaces serve as features predicting interfacial charge recombination rates. ML can predict key outputs like fill factor (FF). Liu et al. employed an ML model using ETL/HTL-perovskite energy level differences and conductivity parameters to predict interfacial recombination rates [67]. Critical findings: Controlling the ETL conduction band minimum (e.g., SnO_2_)-to-perovskite CBM offset to 0.2–0.3 eV and the HTL valence band maximum (e.g., Spiro-OMeTAD)-to-perovskite VBM offset to <0.1 eV reduced interfacial recombination by ~40% and increased FF above 0.8. Comparing n-i-p and p-i-n architectures, the model identified superior energy level alignment in the inverted structure, increasing charge extraction efficiency by ~10% over n-i-p structures. This provides a quantitative basis for designing high-efficiency PSC architectures.

The core logic of ML prediction is that feature engineering defines dimensions, model learning constructs correlations, and multi-scale mapping enhances accuracy. Compared to the trial-and-error experimental method, the key advantage of ML lies in its ability to process hundreds of features simultaneously, uncover hidden correlations overlooked by empirical knowledge, and adapt quickly to new systems via small-sample transfer learning. Future advancements, fueled by accumulating high-throughput experimental data and the development of multimodal models (integrating text, image, and numerical data), promise further breakthroughs in prediction accuracy and applicability. This will accelerate the transition of perovskite materials from the laboratory to industrial-scale production.

### 4.4. Intelligent Optimization of Fabrication Processes

Perovskite solar cell fabrication involves intricate processes where minor parameter deviations can drastically reduce device efficiency. Traditional process optimization methods rely on inefficient trial-and-error; in contrast, machine learning enables targeted and efficient process optimization by identifying key parameters and their correlations.

**Figure 22:**
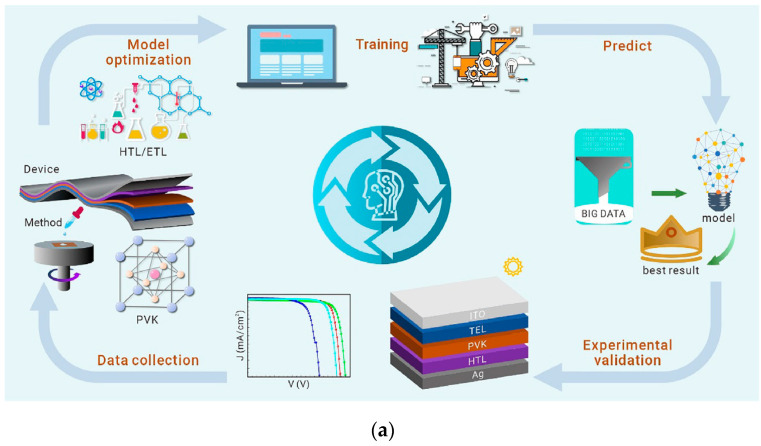

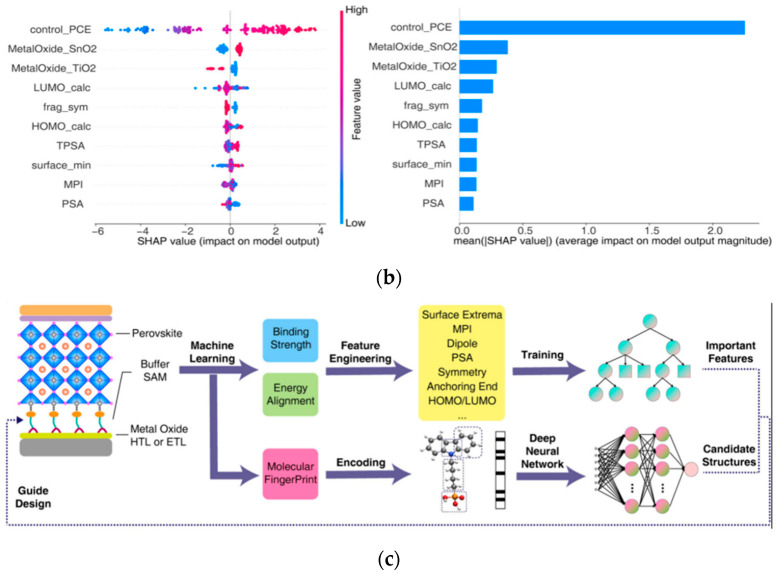
(**a**) The research process of perovskite solar cells combined with machine learning includes four main parts: data collection, model training, predicting target attributes, and experimental verification [68] Copyright 2024, Innovation. Feature importance analysis: (**b**) Important features from the SHAP analysis on the XGBoostmodel, (**c**) workflow of the machine-learning process with combinations of features and fingerprints [70] Copyright 2024, American Chemical Society.

In order to improve the prediction accuracy and precisely control process parameters, Chen et al. developed an ML model incorporating a coarse estimation procedure (CEP) strategy [68]. Using excess non-stoichiometric components (Ensc) and perovskite additive compounds (Pac) as CEP variables significantly improved model performance: the coefficient of determination (R^2^) on the test set increased by 16.14%, while the root mean square error (RMSE) decreased by 20.44%. A stacking model identified key process parameters affecting PCE in order of impact: Ensc amount, perovskite layer thickness, thermal annealing time, perovskite deposition solvent, solvent mixing ratio, and Pac. Experimental validation confirmed that PSCs with 10% excess PbI_2_ exhibited higher PCEs than those with 5% excess, demonstrating the efficacy of Ensc for performance enhancement. The research process are shown in Figure 22a. This work provides vital guidance for optimizing PSC processes, improving performance, and reducing time and costs.

Nagasawa et al. proposed a Random Forest (RF)-based ML approach for screening donor-conjugated molecules in organic photovoltaics [69]. Results indicated the RF classifier outperformed Artificial Neural Networks in predicting polymer PCE, capturing more accurately the association between material features and performance, thereby providing a more reliable tool for identifying highly efficient conjugated molecules.

Gao et al. implemented an ML-driven process parameter optimization strategy [70]. They built a PCE prediction model (PCEPM) using an ensemble of base learners: Random Forest, Extreme Gradient Boosting Regression, and Adaptive Boosting. This PCEPM predicts device efficiency based on process parameters, allowing researchers to quickly identify optimal fabrication conditions for PSC fabrication. Important features from the SHAP analysis and workflow of the machine-learning process are shown in Figure 22b,c. Utilizing this model led to the fabrication of a PSC achieving a PCE of 23.72%, dramatically accelerating research and development efficiency.

ML enables precise directional control. The studies collectively demonstrate the capability of ML to pinpoint critical process variables, quantify their influence, and achieve directional parameter tuning to enhance performance through data-driven closed-loop optimization. This paves a technological pathway combining efficiency and precision for scaling perovskite solar cell manufacturing. ML has established a systematic intelligent optimization scheme by building “process parameter-performance correlation” models, effectively overcoming the inefficiency inherent in traditional trial-and-error approaches.

## 5. Conclusions and Outlook

Over the past 15 years, PSCs have achieved an astonishing leap from basic research to the eve of industrialization. Their single-junction efficiency is approaching the theoretical limit of 29.4%, and the tandem structure has exceeded 30%, showing immeasurable development potential. Currently, the cross-integration of machine learning and materials science is reconstructing the research paradigm, shortening the discovery cycle of new materials from 2 to 3 years to 6 months, and becoming an important driving force for the development of PSCs. This review focuses on the integration of PSCs performance improvement and ML. Section 3 describes the bottleneck of traditional strategies, and Section 4 proves that ML is the key to break through the bottleneck. In the future, it is necessary to further strengthen the data-driven research paradigm.

### 5.1. Main Challenges of PSCs

In just over a decade, PSCs have achieved fruitful results in materials, preparation processes, interface engineering, and stability improvement, with continuous breakthroughs in power conversion efficiency. However, some key technical problems remain unresolved, which serve as significant obstacles to the further development of perovskites into mature and reliable solar cell materials.

#### 5.1.1. Long-Term Stability Issues

PSCs are prone to decomposition under the thermal stress caused by external factors, leading to performance degradation. In high-temperature and high-humidity environments, perovskite materials are easily decomposed, accompanied by ion migration, phase separation, and structural changes, reducing the efficiency and stability of the device. In addition, under long-term illumination, perovskite materials may undergo photo-induced degradation, affecting the long-term performance of the device. Long-term stability remains the main obstacle to commercial applications.

#### 5.1.2. Large Area Preparation and Efficiency Balance

Most laboratory-prepared PSCs currently use the spin-coating method. Although this method can achieve high PCE for devices, issues such as uneven film thickness and increased defects are likely to occur during large-scale production, resulting in efficiency degradation. Processes like blade coating, spray coating, and inkjet printing can be applied to large-scale preparation, but also face problems such as edge effects and uneven thickness, which lead to a decline in device performance. Another solution, vacuum evaporation, is suitable for flexible substrates but faces key challenges such as high costs and large investment in large-scale production equipment.

### 5.2. Future Outlook of PSCs

#### 5.2.1. Strategies for Stability

The development of PSCs still faces challenges in material stability. Although all-inorganic perovskite tandem cells have improved stability to some extent, perovskite materials may still experience performance degradation under combinations of long-term illumination, high temperature, humidity, and other environmental factors, ultimately limiting the operational lifetime of the devices. The preparation process of tandem PSCs is relatively complex, requiring precise control of parameters such as the thickness, crystallization quality, and interface properties of each functional layer. Currently, it is difficult to achieve large-scale and low-cost industrial production.

However, existing mitigating strategies still face challenges such as material compatibility and scalability. For example, issues like efficiency loss due to the reduction in easily decomposable components and the interface resistance between oxide electrolytes and perovskite layers require extensive trial and error. ML can directly identify the optimal material combinations and process parameters by predicting the materials used in PSCs. The research and development of perovskite interface engineering, inverted structures, and all-inorganic structures are exercises in multi-dimensional extensions of “material screening”. ML accelerates this process from the underlying logic through data-driven accurate prediction. Although the research and development and flexible devices have expanded the application scenarios of PSCs, ML is the core driving force for promoting their transition from the laboratory to industrialization. It can not only shorten the material discovery cycle but also deciphers complex multi-factor coupling interactions, providing quantitative solutions for overcoming various technical bottlenecks.

#### 5.2.2. Integration of PSCs and Machine Learning (ML)

Future research needs to further promote the integration of perovskites technology and ML. The interdisciplinary integration is showing irreplaceable value. In particular, advanced computer algorithms combined with intelligent screening of ML can provide more powerful tools for research. The core advantage of perovskite materials lies in their highly tunable chemical composition. Different combinations of elements can significantly affect key properties of the materials, such as the bandgap and carrier mobility. The traditional screening method, which relies on experimental trial and error, often takes months or even years to explore these variations. ML, however, is transforming this process through high-throughput computation and data mining. Researchers can use historical experimental data and density functional theory calculation results as training sets for the model to learn, thereby revealing the potential and hidden relationship between material composition and performance. In addition, machine learning-driven process optimization models are expected to play an increasingly important role in enabling intelligent preparation process of PSCs. More importantly, ML allows for an accurate prediction of device performance. In traditional research on PSCs, even with a fixed material composition, key parameters—such as PCE and long-term operation decay rate—still rely on repeated experimental adjustments. In contrast, a well-trained ML model can directly predict performance outcomes based on the composition, structure, and processing parameters. The integration between perovskites and ML holds substantial promise and is poised to open new frontiers in the field.

In the future, through the exploration of new materials, the development of tandem and flexible PSCs, and breakthroughs in industrialization technologies, PSCs are expected to occupy a significant position in the photovoltaic market and provide robust support for the global clean energy transition. The integration of ML and perovskites research is poised to form an industrial ecosystem marked by “complementary advantages”: perovskites offer an ideal application scenario for ML through their diversified optimization approaches and abundant research data; in turn, ML with its powerful learning capabilities and intelligent analytical methods, can identify optimal development strategies for perovskite development, supporting PSCs in advancing toward higher efficiency and lower costs, collectively propelling the perovskite photovoltaic industry into a new era. With the continued research advancement, we believe that perovskite solar cells, aided by ML, will offer increasingly effective solutions to global energy challenges and pave the way toward a sustainable energy future.

## Data Availability

No new data were created or analyzed in this study. Data sharing is not applicable to this article.

## Abbreviations

The following abbreviations are used in this manuscript:

PSCs	Perovskite solar cells
PCE	Power conversion efficiency
HTL	Hole transport
ML	Machine learning
MA	Methylammonium cation
FA	Formamidinium
Cs	Cesium
CIGs	Copper indium gallium selenide
V_OC_	Open-circuit voltage
J_SC_	Short-circuit current density
ESL	electron selective layer
ITO	indium tin oxide
DEA	diethanolamine
EDAI	Ethylenediamine hydroiodid
FAI	Formamidinium iodide
HTM	Hole transport materials
SAM	Self-assembled monolayer
PDMs	Polydimethylsiloxane
FAIR	Findable, Accessable, Interoperable, Reusable
FF	Fill factor
CEP	Coarse estimation procedure
Ensc	Excess non-stoichiometric components
Pac	Perovskite additive compounds
RMSE	Root mean square error
ANN	Artificial neural networks
PCEPM	PCE prediction model
